# Changes in the diagnosis of glomerular diseases in east China: a 15-year renal biopsy study

**DOI:** 10.1080/0886022X.2018.1537930

**Published:** 2018-11-28

**Authors:** Qin Zhou, Xin Yang, Meifang Wang, Huiping Wang, Jie Zhao, Yan Bi, Xiayue Wang, Jihong Yao, Ying Chen, Chuan Lin, Xishao Xie, Hong Jiang, Jianghua Chen

**Affiliations:** a Kidney Disease Centre, The First Affiliated Hospital, Zhejiang University School of Medicine, Hangzhou, China;; b Kidney Disease Immunology Laboratory, The Third Grade Laboratory, State Administration of Traditional Chinese Medicine of China, Hangzhou, China

**Keywords:** Glomerular disease, membranous nephropathy, IgA nephropathy, renal biopsy, China

## Abstract

**Background:** There have been some gradual changes in the distribution of renal biopsy pathological diagnoses during recent years. This study aimed to show changes in renal disease prevalence in China by investigating 10 patients diagnosed at our Kidney Disease Centre during the last 15 years.

**Methods and results:** All patients aged 15-year-old or older who underwent renal biopsy at the First Affiliated Hospital, Zhejiang University, from 2001 to 2015 were enrolled. There were 5 common types of primary glomerulonephritis: IgA nephropathy (IgA N), membranous nephropathy (MN), mesangial progressive glomerulonephritis (MsPGN), minimal change disease (MCD), and focal segmental glomerulosclerosis (FSGS), which represented 50%, 16.8%, 15.9%, 8.1% and 2.5% of total cases, respectively. IgA nephropathy was the most common type of primary glomerulonephritis (PGN).

**Conclusions:** Our results mostly showed a new trend that the diagnosis of IgA nephropathy was not increasing and the prevalence of membranous nephropathy had increased, becoming the second most common type of primary glomerulonephritis.

**Key POINTS**
- Distinguished with other domestic studies, IgA nephropathy did not show a trend of continuous growth although it still had about the half proportion of PGN, whereas membranous nephropathy kept rising and became the second common PGN.
- Concerning SGN, LN peaked in the younger-age and middle-age groups with a significant female prevalence, DN, BANS and SV had a male predominance peaking in the middle-age and old-age groups.

## Introduction

Diagnostic information obtained from renal biopsy is critical for accurate diagnosis, selection of the most appropriate treatment, and prediction of patient prognosis. In recent years, retrospective studies of renal biopsies, with statistical analyses of different regions, races and time periods, have provided informative findings. For adults, IgA nephropathy is the most common primary glomerulonephritis (PGN) [1–14] in many European countries [1–7], some Asian countries [8–12], the United States [13], and Australia [14]. In addition, the ratio of IgA N continually increased during the previous year [2,7,8,10–13], and studies in Japan [9] and Korea [10] showed that there is no such increasing trend for membranous nephropathy (MN). Furthermore, recent studies from China have shown similar results [11,12]. Now, in our study, the diagnosis of MN has significantly increased during the first 15 years of the 21^st^ century, while IgA N showed an increasing trend in the first 10 years and have started to decrease slowly in the most recent 5 years. Moreover, lupus nephritis (LN) and Henoch-Schonlein nephritis (HSN) remain the most common secondary glomerulonephritis (SGN) while diabetic nephropathy (DN) is less prevalent in our study compared to reports from some Western countries. Therefore, we aimed to evaluate changes in the proportion of renal pathologic diagnoses in East China during the last 15 years.

## Subjects and methods

Overall, 10 877 patients (without renal transplantation) underwent renal biopsy at the First Affiliated Hospital, Zhejiang University during the 15 years period from January 2001 to December 2015. We retrospectively analyzed records of patients aged ≥15 years old. Patients with incomplete records, inadequate biopsies, and repeated biopsies were excluded. In the end, 10 779 cases were included in our analysis. The date of renal biopsy, age, sex, pathologic type and pathologic diagnosis was collected.

Clinical indications for renal biopsy were as follows: (1) nephrotic syndrome or nephrotic range proteinuria, (2) acute nephritic syndrome, (3) rapidly progressive glomerulonephritis, (4) chronic nephritic syndrome, (5) asymptomatic hematuria or proteinuria, and (6) acute or chronic renal failure without definite cause. There was no significant change in the indications for renal biopsy during the observation period. Almost all patients, without obvious contraindications, will have a biopsy as long as they meet the criteria. This study was approved by the local ethics committees. Informed consent was obtained for renal biopsy from each patient.

Data were divided into 3 groups according to 5-year intervals: 2001–2005, 2006–2010 and 2011–2015. Biopsies were also divided into 6 groups according to patients’ age for stratified analysis: 15–24 years old, 25–34 years old, 35–44 years old, 45–54 years old, 55–64 years old, and elderly (≥65 years old). All biopsy specimens were prepared and examined by the same group of clinicians, pathologists and technicians. A renal biopsy was processed for light microscopy, immunofluorescence microscopy and electron microscopy in all submitted cases. The final diagnosis made for each patient was based on both clinical and histologic investigations.

All biopsies were categorized for the purpose of this analysis as PGN, SGN, hereditary nephropathy (HN), tubulointerstitial nephropathy (TIN), or other miscellaneous or undefined histological diagnoses. In this study, we analyzed only the common categories of PGN and SGN. PGN included IgA N, MN, non-IgA mesangial proliferative glomerulonephritis (MsPGN), minimal change disease (MCD), and focal segmental glomerulosclerosis (FSGS). SGN included LN, HSN, DN, systemic vasculitis (SV), hepatitis B virus-associated glomerular nephropathy (HBV-GN), benign nephrosclerosis (BANS) and amyloidosis nephropathy (AMYN).

Results are presented as the means and the standard deviations or the medians with the interquartile ranges, and categorical variables are presented as frequencies with percentages. The distribution of patients with various renal biopsy diagnoses compared between the 5-year intervals, age groups and sex was calculated using Pearson’s chi-square analysis. Bonferroni method was accepted when involving multiple comparisons among groups. All statistical tests were two-sided, with a value for *p* < .05 defined as significant. All statistical analyses were performed using SPSS software, version 22.0 (Chicago, IL).

## Result

**Figure 1.**
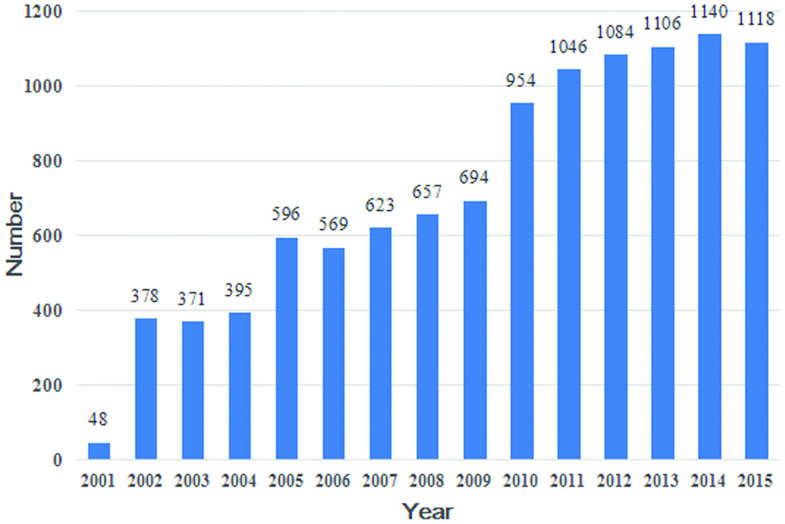
Number of renal biopsies per year.

In total, 10 779 patients were reviewed. Mean patient age at the time of biopsy was 40 ± 14.86 years old ranging from 15 to 87 years. There was a gradual increase in the mean age upon renal biopsy with subsequent 5-year intervals, including 37.07 ± 13.77 years old in 2001–2005, 39.71 ± 14.37 years old in 2006–2010 and 42.98 ± 15.18 years old in 2011–2015. There was a sharp increase in the number of patients who underwent renal biopsy throughout the period of analysis, from less than 50 cases in 2001 to more than 1000 cases during the most recent 5 years (Figure 1).

**Figure 2.**
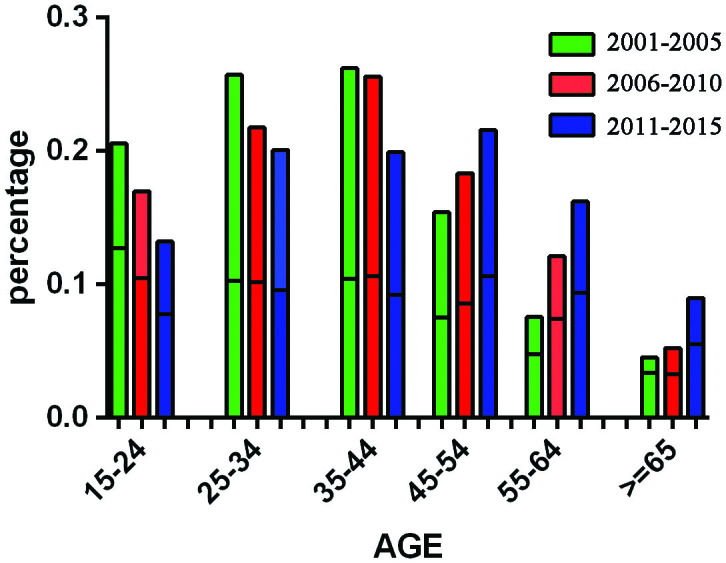
Age- and gender-adjusted distribution rate of renal diagnoses by year.

As shown in Figure 2, the percentage of patients younger than 45 years old consistently declined from the period 2001–2005 to 2011–2015, whereas the proportion of patients over 45 years old significantly increased in the same period. There was a female predominance among patient aged 25–55 years, while in younger and older groups, males were more common.

Overall, PGN remained the most common type of histologic diagnosis, comprising 78.8% (8494/10779) of all biopsies; this included 1478 biopsies (82.7%) from 2001 to 2005, 2794 biopsies (79.9%) from 2006 to 2010 and 4222 biopsies (76.8%) from 2011 to 2015, with a slight decreasing trend over time. To the contrary, the rate of SGN increased from 14.2% in 2001–2005 to 17.4% in 2006–2010 and 20.1% in 2011–2015. The chi-squared test revealed a significant difference in the number of PGN patients among three 5-year intervals (*p* < .001). The diagnosis of TIN and HN were comparable across all three time periods. In total, a significant difference in the sex distribution, the male-to-female ratio was 1.04:1 (Pearson’s *Χ*^2^ 35.7, *p* < .001). Multiple comparisons by Bonferroni method revealed sexy distribution difference of respective pathological pattern. The results showed male predominance among PGN biopsies (1.09:1) and HN biopsies (2.44:1) but a female predominance among SGN biopsies (Table 1).

**Table 1.** Renal biopsy diagnoses by years.

	2001–2005		2006–2010		2011–2015		Pearson's X	*p*	All years	
	*n*	rate	*n*	rate	*n*	rate			*n*	male/female
PGN	1478	82.7%	2794	79.9%	4222	76.8%	31.024	<.001	8494	1.09:1^a^
SGN	253	14.1%	608	17.4%	1106	20.1%	34.927	<.001	1967	0.85:1^a^
TIN	47	2.6%	81	2.3%	144	2.6%	0.903	.637	272	0.97:1
HN	8	0.4%	11	0.3%	12	0.2%	2.598	.273	31	2.44:1^a^
Other	2	0.1%	3	0.1%	10	0.2%	1.539	.463	15	1.50:1^a^
Total	1788	100.0%	3497	100.0%	5494	100.0%	–	–	10779	1.04:1

^a^Significant difference at the 0.05 level in those diagnosed by Chi-squared test. PGN: primary glomerulonephritis; SGN: secondary glomerulonephritis; TIN: tubulointerstitial nephropathy; HN: hereditary nephropathy.

**Figure 3.**
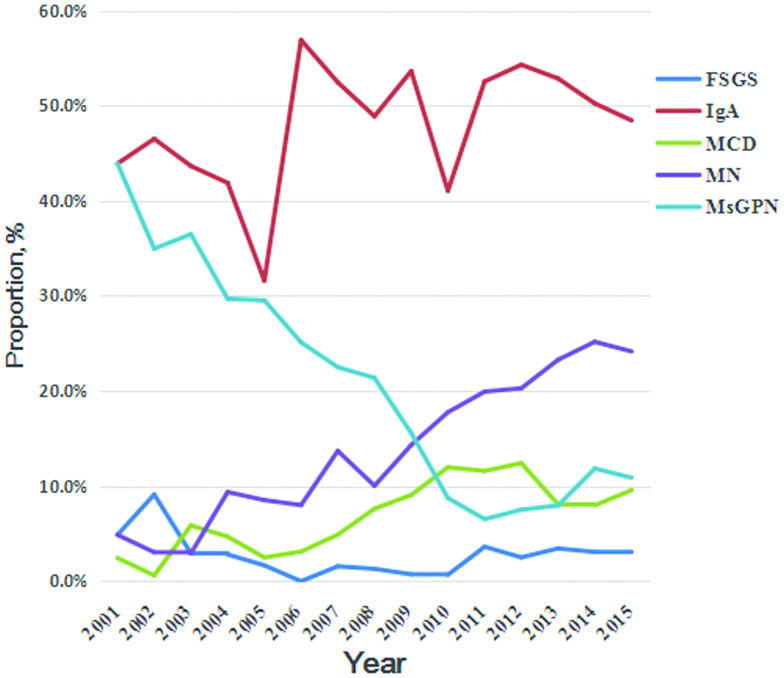
Frequency of specific primary glomerular diseases.

The most frequent diagnosis within the PGN group was IgA N (*n* = 4418, 50%); IgA N showed a gradually decreasing trend in the most recent 5 year time period. The next most common PGN diagnosis was MN (16.8%), followed by MsPGN (15.9%), MCD (8.1%), and FSGS (2.5%). MsPGN was the second most common primary glomerular disease in the initial year (2001), but the rate of MN dramatically increased to account for an equivalent number of biopsies in the middle time period and surpassed all diagnoses except IgA N to become the second most common PGN after 2009. During the same period, the diagnosis of MsPGN decreased significantly, whereas the proportion of biopsies diagnosed with FSGS remained constant over the entire study period. The rate of MCD diagnosis underwent a rapid rise during the middle time period, and the most recent time period showed approximately the same rate of diagnosis. Except MsPGN, other categories of PGN show a distinguished sexy ratio: more female patients in IgA N and more male persons in MN, FSGS and MCD (Table 2, Figure 3).

**Table 2.** The distribution of sex in the common histological categories in PGN.

	Total	Male	Female	*p*
IgA	4418(50%)	2180^a^	2288^b^	<.001
MN	1428(16.8%)	846^a^	582^b^	<.001
FSGS	215(2.5%)	123^a^	92^a^	.132
MCD	685(8.1%)	457^a^	228^b^	<.001
MsPGN	1354(15.9%)	659^a^	695^b^	.005

The same superscript letter denotes a subset of histological categories whose sexy proportion does not differ significantly at the 0.05 level by multiple comparisons in Bonferroni method. MN: membranous nephropathy; FSGS: focal segmental glomerulosclerosis; MCD: minimal change disease; MsPGN: non-IgA mesangioproliferative glomerulonephritis.

**Figure 4.**
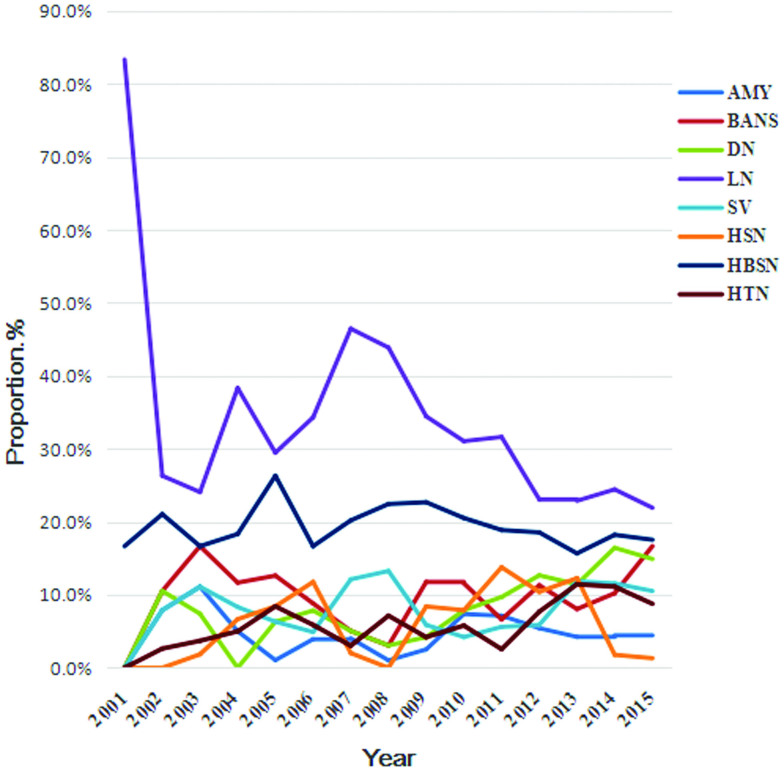
Frequency of specific secondary glomerular diseases.

There were 7 common SGN categories diagnosed at our center: LN (29.2%), HSN (19.2%), DN (9.9%), SV (8.5%), HBV-GN (7.8%), BANS (10.3%) and AMYN (4.8%). There was a male predominance among DN, BANS, AN and HBV-GN biopsies with statistically significant difference whereas females were absolutely prevalent among LN biopsies (Table 3). LN was the most common SGN, accounting for 20%–50% during the whole study period; this diagnosis showed a modest decline after 2008. There was no significant change in the trends of other SGN diagnoses (Figure 4).

**Table 3.** The distribution of sex in the common histological categories in SGN.

	Total	Male	Female	*p*
LN	575(29.2%)	88^a^	487^b^	<.001
HSN	375(19.1%)	190^a^	185^b^	.04
DN	195(9.9%)	137^a^	58^b^	<.001
SV	167(8.5%)	79^a^	88^a^	.705
BANS	203(10.3%)	101^a^	102^a^	.246
AMYN	95(4.8%)	68^a^	27^b^	<.001
HBV-GN	138(7.0%)	95^a^	43^b^	<.001

The same superscript letter denotes a subset of histological categories whose sexy proportion does not differ significantly at the 0.05 level by multiple comparisons in Bonferroni method. LN: lupus nephritis; HSN: Henoch-Schonlein nephritis; DN: diabetic nephropathy; SV: systemic vasculitis; BANS: benign nephrosclerosis; AMYN: amyloidosis nephropathy; HBV-GN: hepatitis B virus-associated glomerular nephropathy.

Table 4 and Table 5 showed that the temporal trends in the renal biopsy frequencies of the most common PGN and SGN subtypes by age strata. As shown in Table 4, the frequency of MN increased significantly (*p* < .001) and nearly increased 2 times from 2006–2010 to 2011–2015. The analysis by age category indicated that the frequency of MN in all age categories increased significantly over time (*p* < 0.001).The distribution of IgA N by age group appeared a ‘triangle’ shape, peaking in the 25–34 years old age group (1302/1943, 67.0%) and 35–44 years old age group (1225/2005, 61.1%), which corresponded with previous reports from Peking University in China [12]. And the LN presented a similar distribution tendency. Overall, compared to IgA N, the age-group predilection of MN was older in the 55–64 and ≥65 category. In contrast, MCD was most commonly diagnosed in the youngest age group of 15–24 category (252/1355, 18.6%), the second common happening in ≥65 category with 12.0% and 55–64 category with 8.6%.

**Table 4.** Temporal trends in the renal biopsy frequencies of the most common PGN subtypes by age strata.

Age	PGN	2001–2005	2006–2010	2011–2015	Total	*p*
category		*n*(%)	*n*(%)	*n*(%)	*n*(%)	
15–24	IgA	134(43.8%)	241(51.72%)	272(46.9%)	647(47.9%)	.081
	MN	4(1.3%)	32(6.87%)	58(10.0%)	94(7.0%)	<.001
	FSGS	9(2.9%)	9(1.93%)	31(5.3%)	49(3.6%)	.010
	MCD	17(5.6%)	75(16.09%)	160(27.6%)	252(18.6%)	<.001
	MsGPN	128(41.8%)	100(21.46%)	43(7.4%)	271(20.0%)	<.001
25–34	IgA	249(61.6%)	442(70.16%)	611(67.3%)	1302(67.0%)	.017
	MN	12(3.0%)	35(5.56%)	79(8.7%)	126(6.5%)	<.001
	FSGS	13(3.2%)	6(0.95%)	28(3.1%)	47(2.4%)	.014
	MCD	13(3.2%)	28(4.44%)	88(9.7%)	129(6.6%)	<.001
	MsGPN	107(26.5%)	103(16.35%)	82(9.0%)	292(15.0%)	<.001
35–44	IgA	214(51.1%)	467(63.97%)	544(63.6%)	1225(61.1%)	<.001
	MN	22(5.3%)	65(8.90%)	143(16.7%)	230(11.5%)	<.001
	FSGS	13(3.1%)	7(0.96%)	17(2.0%)	37(1.8%)	.031
	MCD	6(1.4%)	26(3.56%)	42(4.9%)	74(3.7%)	.008
	MsGPN	123(29.4%)	127(17.40%)	80(9.3%)	330(16.5%)	<.001
45–54	IgA	69(30.8%)	265(52.37%)	434(48.9%)	768(47.5%)	<.001
	MN	25(11.2%)	92(18.18%)	255(28.7%)	372(23.0%)	<.001
	FSGS	11(4.9%)	1(0.20%)	27(3.0%)	39(2.4%)	<.001
	MCD	4(1.8%)	33(6.52%)	35(3.9%)	72(4.5%)	.009
	MsGPN	86(38.4%)	92(18.18%)	83(9.4%)	261(16.1%)	<.001
55–64	IgA	33(30.8%)	116(35.91%)	207(31.6%)	356(32.8%)	.362
	MN	16(15.0%)	91(28.17%)	263(40.2%)	370(34.1%)	<.001
	FSGS	8(7.5%)	1(0.31%)	20(3.1%)	29(2.7%)	<.001
	MCD	4(3.7%)	40(12.38%)	49(7.5%)	93(8.6%)	.006
	MsGPN	26(24.3%)	48(14.86%)	63(9.6%)	137(12.6%)	<.001
>65	IgA	14(21.2%)	36(25.90%)	70(20.8%)	120(22.2%)	.472
	MN	23(34.8%)	56(40.29%)	157(46.7%)	236(43.6%)	.135
	FSGS	4(6.1%)	0(0.00%)	10(3.0%)	14(2.6%)	.029
	MCD	4(6.1%)	17(12.23%)	44(13.1%)	65(12.0%)	.274
	MsGPN	11(16.7%)	24(17.27%)	28(8.3%)	63(11.6%)	.009
Total	IgA	713(48.2%)	1567(56.1%)	2138(50.6%)	4418(52.0%)	<.001
	MN	102(6.9%)	371(13.3%)	955(22.6%)	1428(16.8%)	<.001
	FSGS	58(3.9%)	24(0.9%)	133(3.2%)	215(2.5%)	<.001
	MCD	48(3.2%)	219(7.8%)	418(9.9%)	685(8.1%)	<.001
	MsGPN	481(32.5%)	494(17.7%)	379(9.0%)	1354(15.9%)	<.001

PGN: primary glomerulonephritis; MN: membranous nephropathy; FSGS: focal segmental glomerulosclerosis; MCD: minimal change disease; MsPGN: non-IgA mesangioproliferative glomerulonephritis.

**Table 5.** Temporal trends in the renal biopsy frequencies of the most common SGN subtypes by age strata.

Age	SGN	2001–2005	2006–2010	2011–2015	Total	*p*
category		*n*(%)	*n*(%)	*n*(%)	*n*(%)	
15–24	LN	18(32.7%)	42(38.5%)	50(37.3%)	110(36.9%)	.761
	HSN	24(43.6%)	54(49.5%)	62(46.3%)	140(47.0%)	.755
	DN	0.0%	0.0%	0.0%	–	-
	SV	1(1.8%)	2(1.8%)	2(1.5%)	5(1.7%)	.975
	BANS	1(1.8%)	3(2.8%)	2(1.5%)	6(2.0%)	.780
	AMYN	0.0%	0.0%	0.0%	–	-
	HBS-GN	0.0%	3(2.8%)	14(10.4%)	17(5.7%)	.005
25–34	LN	23(52.3%)	60(50.8%)	80(44.9%)	163(47.9%)	.504
	HSN	8(18.2%)	22(18.6%)	35(19.7%)	65(19.1%)	.963
	DN	1(2.3%)	1(0.8%)	3(1.7%)	5(1.5%)	.753
	SV	3(6.8%)	3(2.5%)	3(1.7%)	9(2.6%)	.164
	BANS	6(13.6%)	8(6.8%)	12(6.7%)	26(7.6%)	.277
	AMYN	0.0%	0.0%	0.0%	–	-
	HBS-GN	1(2.3%)	14(11.9%)	19(10.7%)	34(10.0%)	.177
35–44	LN	25(38.5%)	74(51.4%)	66(30.4%)	165(38.7%)	<.001
	HSN	12(18.5%)	24(16.7%)	28(12.9%)	64(15.0%)	.434
	DN	2(3.1%)	7(4.9%)	24(11.1%)	33(7.7%)	.030
	SV	2(3.1%)	5(3.5%)	5(2.3%)	12(2.8%)	.798
	BANS	11(16.9%)	15(10.4%)	32(14.7%)	58(13.6%)	.351
	AMYN	2(3.1%)	1(0.7%)	4(1.8%)	7(1.6%)	.431
	HBS-GN	3(4.6%)	9(6.3%)	28(12.9%)	40(9.4%)	.038
45–54	LN	10(16.9%)	29(25.9%)	50(19.6%)	89(20.9%)	.286
	HSN	5(8.5%)	13(11.6%)	30(11.8%)	48(11.3%)	.765
	DN	9(15.3%)	13(11.6%)	63(24.7%)	85(20.0%)	.007
	SV	7(11.9%)	8(7.1%)	19(7.5%)	34(8.0%)	.493
	BANS	6(10.2%)	17(15.2%)	42(16.5%)	65(15.3%)	.479
	AMYN	5(8.5%)	3(2.7%)	6(2.4%)	14(3.3%)	.054
	HBS-GN	5(8.5%)	11(9.8%)	18(7.1%)	34(8.0%)	.660
55–64	LN	3(9.7%)	15(17.2%)	20(10.4%)	38(12.2%)	.240
	HSN	3(9.7%)	9(10.3%)	26(13.5%)	38(12.2%)	.686
	DN	1(3.2%)	11(12.6%)	38(19.7%)	50(16.1%)	.040
	SV	4(12.9%)	18(20.7%)	39(20.2%)	61(19.6%)	.609
	BANS	6(19.4%)	7(8.0%)	19(9.8%)	32(10.3%)	.194
	AMYN	2(6.5%)	12(13.8%)	21(10.9%)	35(11.3%)	.521
	HBS-GN	2(6.5%)	1(1.1%)	6(3.1%)	9(2.9%)	.306
>65	LN	0.0%	4(10.5%)	6(4.7%)	10(5.6%)	.266
	HSN	2(16.7%)	3(7.9%)	15(11.7%)	20(11.2%)	.667
	DN	1(8.3%)	4(10.5%)	17(13.3%)	22(12.4%)	.820
	SV	3(25.0%)	9(23.7%)	34(26.6%)	46(25.8%)	.936
	BANS	2(16.7%)	3(7.9%)	11(8.6%)	16(9.0%)	.624
	AMYN	4(33.3%)	10(26.3%)	25(19.5%)	39(21.9%)	.413
	HBS-GN	0.0%	1(2.6%)	1(0.8%)	2(1.1%)	.592
Total	LN	79(31.2%)	224(36.8%)	272(24.6%)	575(29.2%)	<.001
	HSN	54(21.3%)	125(20.6%)	196(17.7%)	375(19.1%)	.220
	DN	14(5.5%)	36(5.9%)	145(13.1%)	195(9.9%)	<.001
	SV	20(7.9%)	45(7.4%)	102(9.2%)	167(8.5%)	.406
	BANS	32(12.6%)	53(8.7%)	118(10.7%)	203(10.3%)	.191
	AMYN	13(5.1%)	26(4.3%)	56(5.1%)	95(4.8%)	.745
	HBS-GN	11(4.3%)	39(6.4%)	86(7.8%)	136(6.9%)	.129

LN: lupus nephritis; HSN: Henoch-Schonlein nephritis; DN: diabetic nephropathy; SV: systemic vasculitis; BANS: benign nephrosclerosis; AMYN: amyloidosis nephropathy; HBV-GN: hepatitis B virus-glomerular nephropathy.

LN, HSN, DN, SV, BANS, AMYN and HBS-GN were the most common SGN diagnoses. As shown in Table 5, LN peaked in the age 25–34 category. HSN peaked in the age 15–24 category and decreased sharply in the succeeding age categories before stabilizing around age 45 and beyond. HBS-GN peaked in the age 25–34 category. DN and BANS both peaked in the middle-age groups, between the age of 45 and 54 years old, whereas the diagnosis of AMYN appeared mostly in older age groups. The diagnosis of HBV-GN was most common in the 25–34 category, with a male predominance, consistent with previously reported age and sex distributions from China [15]. This is related to the age of onset of hepatitis B patients in China. The frequencies of SV and AMYN increased with age. The constituent ratio of each SGN changed dynamically at different time intervals. LN decreased significantly (*p* < .001), DN increased over twice (*p* < .001).

**Figure 5.**
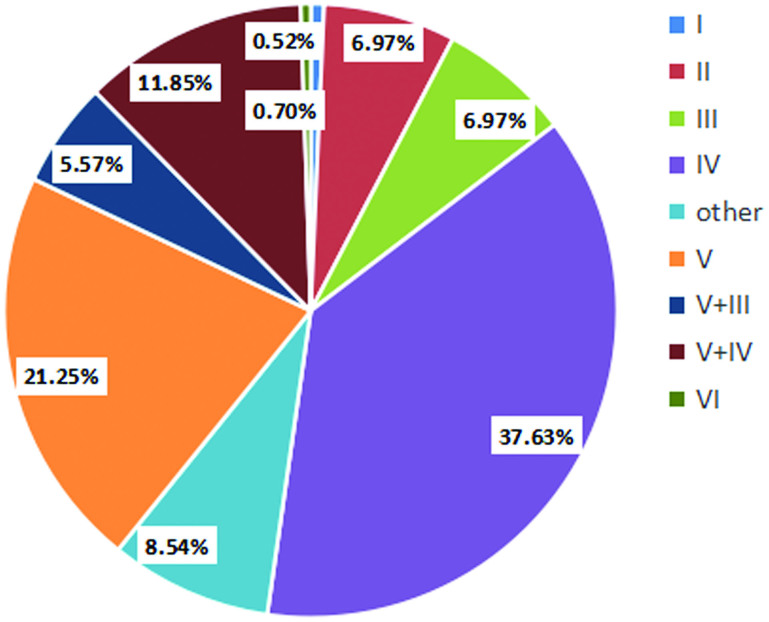
Proportion of lupus nephritis diagnoses.

Type IV and type V were the most common histologic diagnoses within LN, both types in total occupying about 60%. The following was IV + V miscellaneous type (11.9%). As for other types, each type respectively was less than 10% with a small share (Figure 5).

## Discussion

There was a dramatic increase in the number of renal biopsies at our center during the past 15 years, which correlates with an increased number of ward beds and improvements in renal biopsy technology. In addition, with improvements in economic status and health care reform, more patients could afford payment for renal biopsy. Based on the stratified analysis of age, the proportion of biopsies in patients over 45 years old increased, consistent with the recent report on the prevalence of biopsy-proven renal diseases in elderly Chinese patients from the Nan Jing [16]. The increasing proportion of biopsies among the elderly might be due to aging of the Chinese population, as well as improved technology allowing for safe renal biopsy over a larger age range.

We found IgA N to be the most common PGN, comprising about 50% of cases of PGN; this is similar to previous reports from our country [11,12,17]. However, there is a significant difference in the distribution of the most frequent PGN among different regions and races. For example, in many European countries [1–7], some Asian countries [8–12], the United States [13], and Australia [14], IgA N is the most common PGN; in contrast, the most common PGN is FSGS in Brazil [18–20], India [21], and the United States [22–26], MsPGN in South Africa [27] and Demark [28], MN in Macedonia Serbia [29,30], membranoproliferative glomerulonephritis (MPGN) in Romania [31], and IgM nephropathy in Thailand [32]. The variability above noted may represent differences in the regions, environmental level and genetic background influencing patients’ susceptibility to various nephropathy categories. Furthermore, the difference in indications for renal biopsy in various parts of the world is the major factor contributing to diversity in distribution of renal diseases in various countries. Concerning IgA N, we found a slow decline starting in 2010, which is different from the increasing trend found in previous domestic reports [11,12]. This natural, social and lifestyle change may be due to economic and industrial development in China. A similar finding was also observed in other Chinese subpopulations [33]. To some extent, the decrease in the biopsies from the young age group to the middle age group with subsequent increase in the older age group might reflect a higher susceptibility to MN among older patients. The fact also illustrated MN has kept an increasing trend. In addition, we think IgAN is a disease of an early onset age and a long course. With the extensive development of medical treatment in China, the time to discover and treat patients is gradually advanced, that making the new cases tend to decline steadily. However, because we identified the trend of IgA N decreasing over a recent 5-year time period, it was difficult to determine if this is a long-term trend, an anomaly or just a temperate wave; further long-term observation is needed.

Another significant difference we found was that MN dramatically increased during the study period, representing a larger proportion (25.2%) of PGN in 2014 than in the other years. We found a similar trend reported in previous studies in China [34]. The result of our study may be interpreted in the context of increasingly aggressive biopsy performance; however, this is unlikely to be the main reason for the increasing incidence of MN, because there has been no increase in the rate of biopsy performance in the most recent 5 years, and the rate of MN diagnosis has continued to rise. The increased proportion of MN diagnoses could be due to aging of the Chinese population. A recent report from Nanjing [16] has shown that MN was the most common histologic diagnosis in patients over 65 years old. We found that MN surpassed IgA N to become the most common PGN in the 55– 64-year-old age group. In addition, there are some case reports that susceptibility to MN correlates with heavy metal exposure and accumulation, including argentum [35], mercury [36], lithium [37], and others. Thus the increased rate of MN might be related to increased pollution due to greater use of automobiles and industrialization. Recent studies have shown that long-term exposure to high levels of PM25 may be associated with an increased risk of MN [38]. However, the specific mechanism still needs further study.

There was a noticeable decrease in the diagnosis of MsPGN during the first decade of the 21^st^ century, which continued through the most recent 5 years. As we know MsPGN is an early stage of many diseases, we are currently used for a small amount of proteinuria or/and hematuria, that with certain mesangial cells and mesangial matrix hyperplasia but without a dense substance by LM and EM. And as the disease diagnosis progresses, the number of diagnoses of this disease in our center is also gradually decreasing. This is similar to previous domestic reports [11,12,17]. One reason for this phenomenon is that many cases were diagnosed as other categories, due to improvements in diagnosis and microscopic analysis.

Moreover, we found a higher proportion of LN and a lower proportion of FSGS and DM compared with European [1–7] and American [13,22–26] reports, partly due to differences in heredity and the environment. However, variation in the frequency of DM could be explained by other factors. Considering renal biopsy indications, we did not have any patients underwent renal biopsy for diabetes, except those with unsatisfactory clinical curative effect and those suspected to have a different renal disease. So the prevalence of DN might be underestimated, actually.

There are some limitations of our study. Firstly, as a single-center study, the enrolled patients may not have been an adequate representation of the entire Chinese population. Our hospital is the referral medical center for renal disease in the Zhejiang Province in the eastern part of China, we received many referral patients, particularly severely ill patients. Thus a detection bias was unavoidable. It is also worth mentioning that this study only reflects the frequencies of kidney diseases among those who have undergone renal biopsy, and thus these findings may not be applicable to the general population. However, the reporting of renal biopsy data from a single center with stable biopsy and diagnostic criteria is useful for the assessment of changing patterns in renal disease. Secondly, as it is an observational study, we could only observe the changes in the frequencies of kidney diseases and thus could not fully explain the reasons behind these changes. Some studies are limited by a lack of clinical details and variability in renal biopsy practices across the whole country; we could not determine whether the changes in the proportion of renal biopsy diagnoses were due to differences of environment, patient-specific factors, social development, or differences amongst geographic regions. Hence, follow-up studies are required to elucidate the roles of various factors in the frequencies of kidney diseases.

In conclusion, the pathologic spectrum of renal disease has changed within the last 15 years, especially concerning PGN. IgA N remains the most common PGN, although there was a steady decline in the most recent 5 years. In the 5-year interval comparison, the relative frequency of MN has continued to increase, while the relative frequency of MsPGN has dramatically decreased over the past 15 years. Concerning SGN, LN peaked in the younger-age and middle-age groups with a significant female prevalence, whereas DN, BANS and SV had a male predominance peaking in the middle-age and old-age groups.

## References

[1] Richards NT, Darby S, Howie AJ. et al. Knowledge of renal histology alters patient management in over 40% of cases. Nephrol Dial Transplant. 1994;9:1255–1259. 7816285

[2] Stratta P, Segoloni GP, Canavese C Incidence of biopsy-proven primary glomerulonephritis in an Italian province. Am J Kidney Dis. 1996;27:631–639. 8629621 10.1016/s0272-6386(96)90096-7

[3] Schena FP The Italian Group of Renal Immunopathology. Survey of the Italian Registry of Renal Biopsies. Frequency of the renal diseases for 7 consecutive years. Nephrol Dial Transplant. 1997;12:418–426. 9075118 10.1093/ndt/12.3.418

[4] Rivera F, López-Gómez JM, Pérez-García R Frequency of renal pathology in Spain 1994-1999. Nephrol Dial Transplant. 2002;17:1594–1602. 12198210 10.1093/ndt/17.9.1594

[5] Simon P, Ramee M-P, Boulahrouz R, et al. Epidemiologic data of primary glomerular diseases in western France. Kidney Int. 2004;66:905–908. 15327379 10.1111/j.1523-1755.2004.00834.x

[6] Rychlik I, Jancova E, Tesar V, et al. The Czech registry of renal biopsies. Occurrence of renal diseases in the years 1994-2000. Nephrol Dial Transplant. 2004;19:3040–3049. 15507479 10.1093/ndt/gfh521

[7] Hanko JB, Mullan RN, O’Rourke DM, et al. The changing pattern of adult primary glomerular disease. Nephrol Dial Transplant. 2009;24:3050–3054. 19487734 10.1093/ndt/gfp254

[8] Woo KT, Chiang GS, Pall A, et al. The changing pattern of glomerulonephritis in Singapore over the past two decades. Clin Nephrol. 1999;52:96–102. 10480220

[9] Research Group on Progressive Chronic Renal Disease. Nationwide and long-term survey of primary glomerulonephritis in Japan as observed in 1,850 biopsied cases. Nephron. 1999;82:205–213. 10395992 10.1159/000045404

[10] Chang JH, Kim DK, Kim HW, et al. Changing prevalence of glomerular diseases in Korean adults: a review of 20 years of experience. Nephrol Dial Transplant. 2009;24:2406–2410. 19264742 10.1093/ndt/gfp091

[11] Li LS, Liu ZH Epidemiologic data of renal diseases from a single unit in China: analysis based on 13,519 renal biopsies. Kidney Int. 2004;66:920–923. 15327382 10.1111/j.1523-1755.2004.00837.x

[12] Zhou F, Zhao M, Zou W, et al. The changing spectrum of primary glomerular diseases within 15 years: a survey of 3331 patients in a single Chinese centre. Nephrol Dial Transplant. 2008;24:870–876. 18940885 10.1093/ndt/gfn554

[13] Swaminathan S, Leung N, Lager DJ, et al. Changing incidence of glomerular disease in Olmsted County, Minnesota: a 30-year renal biopsy study. Clin J Am Soc Nephrol. 2006;1:483–487. 17699249 10.2215/CJN.00710805

[14] Briganti EM, Dowling J, Finlay M, et al. The incidence of biopsy-proven glomerulonephritis in Australia. Nephrol Dial Transplant. 2001;16:1364–1367. 11427626 10.1093/ndt/16.7.1364

[15] Wang YY, Zhang W, Zhang Z, et al. A community-based seroepidemiological survey of hepatitis B among adults in Chaoyang district. Chin J Epidemiol. 2015;36:1104–1108. 26837354

[16] Jin B, Zen CH, Ge YC, et al. The spectrum of biopsy-proven kidney diseases in elderly Chinese patients. Nephrol Dial Transplant. 2014;29:2251–2259. 25034755 10.1093/ndt/gfu239

[17] Jin-Hua H, Hui-Xian Z, Min-Lin Z, etet al. Changes in the spectrum of kidney diseases: an analysis of 40,759 biopsy-proven cases from 2003 to 2014 in China. Kidney Dis. 2018;4:10–19. 10.1159/000484717 PMC5848489 29594138

[18] Bahiense-Oliveira M, Saldanha LB, Mota EL, et al. Primary glomerular diseases in Brazil (1979–1999: is the frequency of focal and segmental glomerulosclerosis increasing?. Clin Nephrol. 2004;61:90–97. 14989627 10.5414/cnp61090

[19] Malafronte P, Mastroianni-Kirsztajn G, Betonico GN, et al. Paulista registry of glomerulonephritis: 5-year data report. Nephrol Dial Transplant. 2006;21:3098–3310. 16968733 10.1093/ndt/gfl237

[20] Maria GP, Luiz AR, Gianna MK, et al. An overview on frequency of renal biopsy diagnosis in Brazil: clinical and pathological patterns based on 9617 native kidney biopsies. Nephrol Dial Transplant. 2010;25:490–496. 19633091 10.1093/ndt/gfp355

[21] Narasimhan B, Chacko B, John GT, et al. Characterization of kidney lesions in Indian adults: towards a renal biopsy registry. J Nephrol. 2006;19:205–210. 16736422

[22] Haas M, Spargo BH, Coventry S Increasing incidence of focalsegmental glomerulosclerosis among adult nephropathies: a 20-year renal biopsy study. Am J Kidney Dis. 1995;26:740–750. 7485126 10.1016/0272-6386(95)90437-9

[23] Korbet SM, Genchi RM, Borok RZ, et al. The racial prevalence of glomerular lesions in nephrotic adults. Am J Kidney Dis. 1996;27:647–651. 8629623 10.1016/s0272-6386(96)90098-0

[24] Haas M, Meehan SM, Karrison TG, et al. Changing etiologies of unexplained adult nephritic syndrome: a comparison of renal biopsy findings from 1976–1979 and 1995-1997. Am J Kidney Dis. 1997;30:621–631. 9370176 10.1016/s0272-6386(97)90485-6

[25] Braden GL, Mulhern JG, O'Shea MH, et al. Changing incidence of glomerular diseases in adults. Am J Kidney Dis. 2000;35:878–883. 10793022 10.1016/s0272-6386(00)70258-7

[26] Dragovic D, Rosenstock JL, Wahl SJ, et al. Increasing incidence of focal segmental glomerulosclerosis and an examination of demographic patterns. CN. 2005;63:1–7. 10.5414/cnp63001 15678691

[27] Ikechi O, Charles S, Maureen D, et al. Patterns of renal disease in Cape Town South Africa: a 10-year review of a single-centre renal biopsy database. Nephrol Dial Transplant. 2011;26:1853–1861. 20980357 10.1093/ndt/gfq655

[28] Heaf J, Lokkegaard H, Larsen S The epidemiology and prognosis of glomerulonephritis in Denmark 1985–1997. Nephrol Dial Transplant. 1999;14:1889–1897. 10462267 10.1093/ndt/14.8.1889

[29] Polenakovic MH, Grcevska L, Dzikova S The incidence of biopsyproven primary glomerulonephritis in the Republic of Macedonia long- term follow-up. Nephrol Dial Transplant. 2003;18:26–27. 10.1093/ndt/gfg1039 12817063

[30] Naumovic R, Pavlovic S, Stojkovic D, et al. Renal biopsy registry from a single centre in Serbia: 20 years of experience. Nephrol Dial Transplant. 2008;24:877–885. 18927123 10.1093/ndt/gfn564

[31] Covic A, Schiller A, Volovat C, et al. Epidemiology of renal disease in Romania: a 10 year review of two regional renal biopsy databases. Nephrol Dial Transplant. 2006;21:419–424. 16249204 10.1093/ndt/gfi207

[32] Parichatikanond P, Cawanasuntorapoj R, Shayakul C, et al. An analysis of 3,555 cases of renal biopsy in Thailand. J Med Assoc Thai. 2006;29:106–111. 17044461

[33] Tang L, Yao J, Kong X, et al. Increasing prevalence of membranous nephropathy in patients with primary glomerular diseases: a cross-sectional study in China. Nephrology (Carlton). 2017;22:168–173. 26854278 10.1111/nep.12739

[34] Zhu P, Zhou F-d, Wang S-x, et al. Increasing frequency of idiopathic membranous nephropathy in primary glomerular disease: a 10-year renal biopsy study from a single Chinese nephrology centre. Nephrology (Carlton). 2015;20:560–566. 26086701 10.1111/nep.12542

[35] Watanabe Y, Eguchi A, Kamio M, et al. Case of membranous nephropathy associated with argyria. Nihon Jinzo Gakkai Shi. 2005;47:547–551. 16130411

[36] Serhat A, Olive G, Bernd K, et al. Membranous nephropathy from exposure to mercury in the fluorescent-tube-recycling industry. Nephrol Dial Transplant. 2001;16:2253–2255. 11682678 10.1093/ndt/16.11.2253

[37] Gunjeet K, Muniza M Lithium-induced membranous glomerulonephropathy in a pediatric patient. Pediatr Nephrol. 2009;24:2267–2269. 19582480 10.1007/s00467-009-1245-3

[38] Xu X, Wang G, Chen N, et al. Long-term exposure to air pollution and increased risk of membranous nephropathy in China. J Am Soc Nephrol. 2016;27:3739–3746. 27365535 10.1681/ASN.2016010093 PMC5118492

